# Chronology of tRNA structural dynamics prior to and during interaction with a pseudouridine synthase

**DOI:** 10.1261/rna.080869.125

**Published:** 2026-07

**Authors:** Emily M. Dennis, Nico E. Conoan Nieves, Madison Kadrmas, Abigail L. Vaaler, Margaret L. Barry, David M. Garcia, Julia R. Widom

**Affiliations:** 1 Institute of Molecular Biology, University of Oregon, Eugene, Oregon 97403, USA; 2 Department of Chemistry and Biochemistry, University of Oregon, Eugene, Oregon 97403, USA; 3 Department of Biology, University of Oregon, Eugene, Oregon 97403, USA

**Keywords:** tRNA, RNA modification, pseudouridine, Pus4, smFRET, tRNA structure

## Abstract

Transfer RNA has long served as an exemplar of a thermodynamically stable, structured RNA. Yet, it undergoes significant structural changes upon binding and catalysis by diverse modification enzymes. We leveraged optical binding assays and single-molecule FRET to observe the structural dynamics of two yeast tRNAs, in isolation, and upon interaction with the conserved pseudouridine synthase Pus4/TruB. We show that unmodified and pseudouridylated tRNA^eMet(CAU)^ and tRNA^Thr(AGU)^ all sample open, compact, and intermediate conformations, though tRNA^Thr(AGU)^ exhibits faster dynamics. Consistent with its role in modifying RNAs with different structural properties, Pus4 binds robustly to unmodified tRNA, tRNA that was pseudouridylated prior to engaging Pus4 (“premodified”), and even an unrelated riboswitch RNA. Pus4 binding to tRNA^eMet(CAU)^ leads to additional tRNA conformational states. The ensemble of conformations explored by Pus4-bound premodified tRNA resolved within minutes back into open, compact, and intermediate states. tRNA^eMet(CAU)^ that is initially unmodified more gradually approached the ensemble of structures that were attained rapidly by premodified tRNA. Thus, Pus4 both catalyzes a lasting chemical change on tRNA and remodels it over time after catalysis, perhaps to promote subsequent steps of tRNA maturation.

## INTRODUCTION

**FIGURE 1.**
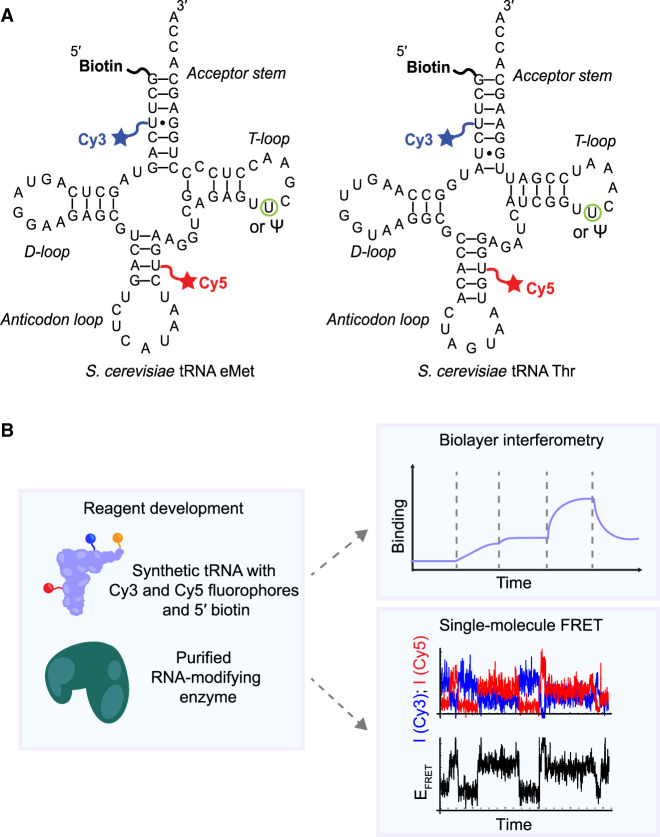
tRNA sequences and methodological approach to investigate how an RNA-modifying enzyme influences tRNA conformation. (*A*) Predicted secondary structures of the tRNA sequences used in this study, including the positions of biotin, Cy3, Cy5, and pseudouridine modification. The four structural motifs are labeled on each tRNA in italics (acceptor stem, T-loop, anticodon loop, and D-loop). (*B*) Summary of experimental approach and workflow.

Pseudouridine (Ψ) is the most abundant RNA chemical modification in nature, and transfer RNAs (tRNAs; Fig. 1A) represent the most common pseudouridylated substrate. tRNAs are formed from four structural motifs that fold onto each other in three dimensions (Giegé et al. 2012). The acceptor stem at one end of a tRNA is sparsely modified and serves as the attachment point for an amino acid. The anticodon stem–loop (ASL) at the opposite end is frequently modified at multiple positions and is involved in mRNA decoding (Krutyhołowa et al. 2019). The heavily modified D-loop and T-loop interact to form the “elbow” where they impart flexibility to the molecule to promote multiple steps of tRNA biogenesis and protein synthesis, including translocation through the ribosome (Zhang and Ferré-D'Amaré 2016). While many sites of pseudouridylation are found in tRNA, position 55 in the T-loop is pseudouridylated in nearly all tRNAs and is most commonly catalyzed by a pseudouridine synthase from the “TruB” family (Rintala-Dempsey and Kothe 2017). Bacteria contain a single enzyme, TruB, that is responsible for all tRNA U_55_ catalysis. Yeast also contain a single enzyme responsible for U_55_ catalysis, Pus4, which is found in the nucleus, cytoplasm, and mitochondria (Becker et al. 1997). Humans encode two paralogous enzymes, TRUB1 and TRUB2, which are found primarily in the nucleus and mitochondria, respectively. In humans, a third enzyme belonging to a different synthase family, Pus10, is located in the cytoplasm and also modifies many tRNAs at U_55_ (Song et al. 2020; Mukhopadhyay et al. 2021). This change from U_55_ to Ψ_55_ creates an additional hydrogen bond donor, increasing base-stacking and helix rigidity, while promoting tertiary interactions between the T-arm and D-arm in the “elbow” region of a folded tRNA. Thus, Ψ_55_ contributes to the characteristic three-dimensional structure of tRNA (Robertus et al. 1974; Shi and Moore 2000; Zhang and Ferré-D'Amaré 2016; Lorenz et al. 2017; Biela et al. 2023). In the budding yeast *Saccharomyces cerevisiae*, Pus4 pseudouridylates 41 out of 42 cytosolic tRNA isoacceptors (Shaw et al. 2024), and 23 out of 24 mitochondrial tRNA isoacceptors (Reinsch and Garcia 2025). In *Escherichia coli*, all tRNAs are pseudouridylated by TruB (Cappannini et al. 2024). Therefore, the majority of tRNA molecules in these species are bound and modified by Pus4/TruB, making this enzyme a useful model for understanding the interplay between tRNA structure and dynamics and an enzyme that chemically modifies it.

Previous studies have identified various RNA sequences and/or structures important for binding and modification by various pseudouridine synthases (Lecointe et al. 1998; Motorin et al. 1998; Ansmant et al. 2000, 2001; Behm-Ansmant et al. 2003; Roovers et al. 2006; Gurha et al. 2007; Gurha and Gupta 2008; Urban et al. 2009; Zhou et al. 2011; Carlile et al. 2014, 2019; Tillault et al. 2018; Purchal et al. 2022; Grünberg et al. 2023; Lin et al. 2024; Schaening-Burgos et al. 2024), including Pus4/TruB (Gu et al. 1998; Phannachet and Huang 2004; Mukhopadhyay et al. 2021). Yet binding by these enzymes can remodel RNAs in ways distinct from the lasting chemical change that they catalyze. An early study on bacterial TruB noted that growth phenotypes associated with its absence could not be explained by its catalytic activity alone (Gutgsell et al. 2000). Subsequently, a second role was more clearly established: That it aids tRNA folding and functions as a tRNA chaperone (Keffer-Wilkes et al. 2016). Other studies have also demonstrated RNA-chaperone-like roles for other tRNA modifying enzymes (Johansson and Byström 2002; Copela et al. 2006; Gc et al. 2020; Keffer-Wilkes et al. 2020). Thus, to exert their full function, tRNA modifying enzymes should (1) bind tRNA, (2) catalyze tRNA chemical modification, and, in some cases, (3) remodel tRNA structure. These three components could each independently affect the dynamics of tRNA structure, or do so interdependently. We sought to clarify these activities by addressing the following questions: (1) Do structural ensembles of unmodified tRNA and pseudouridylated tRNA differ? (2) How does binding by Pus4 affect the distribution of tRNA structures over time? (3) Does the outcome depend on whether the tRNA is pseudouridylated prior to engagement with Pus4?

To address these questions, we leveraged single-molecule Förster resonance energy transfer (smFRET) to track the conformational fluctuations of tRNA and biolayer interferometry (BLI) to monitor association of Pus4 to tRNA (Fig. 1B). We show that Pus4 binds rapidly to both unmodified (U_55_) and pseudouridylated (Ψ_55_, hereafter also referred to as “premodified”) tRNAs, and subsequently navigates unmodified tRNA through a pathway of catalysis and remodeling lasting tens of minutes, which is partially bypassed by premodified tRNA. This pathway involves a temporary population of conformations not prevalent for either U_55_ or Ψ_55_ tRNA in the absence of enzyme. Thus our results show that a prototypical RNA-modifying enzyme shapes the chronology of tRNA structural dynamics through both its catalysis and remodeling.

## RESULTS

### Recombinant Pus4 pseudouridylates yeast tRNAs in vitro

**FIGURE 2.**
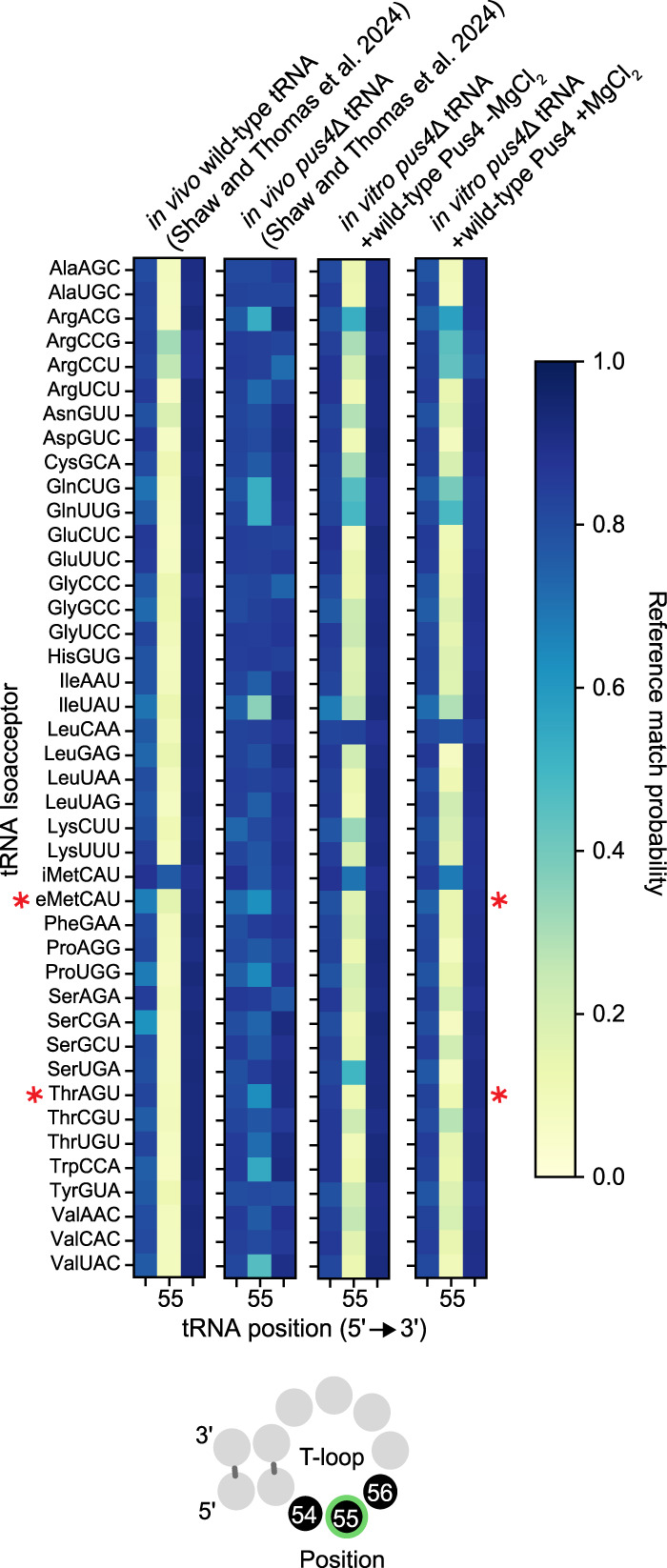
Nanopore direct RNA sequencing shows in vitro modification activity of purified Pus4. Heat maps represent the sequenced nucleotide positions 54–56 of the 42 yeast cytosolic tRNA isoacceptors for tRNA harvested from *pus4*Δ cells and reacted in vitro with purified Pus4, with or without 4 mM MgCl_2_. Results from in vivo tRNA sequencing are from Shaw et al. (2024). Reference match probability corresponds to the probability that a base-called nucleotide agrees with the reference unmodified nucleotide (A, G, C, or U). More frequently miscalled positions, such as pseudouridine at position 55, produce light yellow squares. When position 55 is base-called as a uridine, the square is dark blue. Red stars indicate the cellular tRNAs corresponding to synthetic tRNAs investigated in this study. At the *bottom* is a diagram of a generic yeast tRNA T-loop with black circles denoting bases shown in heat maps. Note that “iMet” refers to the initiator tRNA that decodes the start codon and is different from “eMet,” which is the tRNA that decodes subsequent methionine codons.

We first established a protocol to express and purify *S. cerevisiae* Pus4 protein from *E. coli* cells (Supplemental Fig. S1A). While we attempted to prepare untagged Pus4 protein, it showed a strong propensity to aggregate after tag cleavage, so we proceeded with a GST-tagged full-length version instead, which remained soluble throughout purification. To verify the biochemical activity of the purified protein, we tested its catalysis in vitro (Fig. 2). Using direct RNA sequencing (DRS), a method we previously established to investigate tRNA modification in vivo (Shaw et al. 2024), Ψ_55_ was newly detected on nearly all tRNAs isolated from a *pus4*Δ yeast strain following a 2 min incubation with an excess of purified Pus4. Modification levels were qualitatively comparable to those previously observed in tRNA isolated from WT strains (Shaw et al. 2024). While some differences in pseudouridine levels were observed between the in vivo and in vitro data—most notably a lack of modification of tRNAs Arg^ACG^ and Leu^CAA^ in vitro—the data supported near-full catalytic activity of purified Pus4 on the vast majority of these cell-derived yeast tRNAs. Robustly modified tRNAs included the two employed in this study (details below), tRNA^eMet(CAU)^ (the elongator methionine tRNA, as opposed to the initiator methionine tRNA, tRNA^iMet(CAU)^) and tRNA^Thr(AGU)^, hereafter called tRNA eMet and tRNA Thr, respectively.

### Synthetic modified tRNAs enable binding and smFRET studies

Having established recombinant Pus4 with desired enzymatic activities on the majority of yeast isoacceptors in vitro, we chose two as models for our biophysical studies of RNA binding by Pus4 (using BLI) and tRNA conformational fluctuations (using smFRET). The yeast tRNA eMet and tRNA Thr were chosen for several reasons: (1) both contain uridines at positions 4 and 41, enabling us to use fluorophore-labeling sites and chemistry matching those of several prior studies that performed FRET analysis of tRNA structure (Voigts-Hoffmann et al. 2007; Kobitski et al. 2008, 2011; Dammertz et al. 2011), (2) both have a minimum free energy structure, as predicted by RNAfold (Gruber et al. 2008), that closely resembles the tRNA cloverleaf secondary structure, theoretically reducing formation of noncanonical, off-pathway structures in vitro, and (3) prior evidence demonstrates that both tRNAs are pseudouridylated by Pus4 efficiently in vivo (Shaw et al. 2024), in addition to our work herein that demonstrated their efficient pseudouridylation by Pus4 in vitro (Fig. 2). The tRNAs were labeled with the FRET donor Cy3 at the carbon-5 position of U_4_ in the acceptor stem and with the FRET acceptor Cy5 at the carbon-5 position of U_41_ in the anticodon stem. They were additionally biotinylated at the 5′ end to enable immobilization of the molecules on a microscope slide for smFRET or on a probe for BLI. We prepared two versions each of tRNA eMet and tRNA Thr, containing either U or Ψ at position 55.

In previous work of ours and others, it has been observed that the addition of Ψ_55_ can promote the addition of other T-loop modifications, specifically m^5^U_54_ and m^1^A_58_ (Barraud et al. 2019). We previously identified a subset of yeast isoacceptors in which the presence of Ψ_55_ promoted m^1^A_58_ catalysis in vivo (Shaw et al. 2024). While both tRNA eMet and tRNA Thr used here do exhibit this pattern in vivo, and thus would have also represented interesting models for investigating how multiple T-loop modifications influence tRNA conformation, technical considerations precluded our ability to include m^1^A_58_ in our synthetic sequences. The m^1^A modification undergoes spontaneous conversion to m^6^A via the Dimroth rearrangement (Macon and Wolfenden 1968) under a number of different experimental conditions, including polyacrylamide gels that we used here for RNA purification, which was previously documented in mass spectrometry data (Shaw et al. 2024).

### Pus4 binds rapidly to tRNA

**FIGURE 3.**
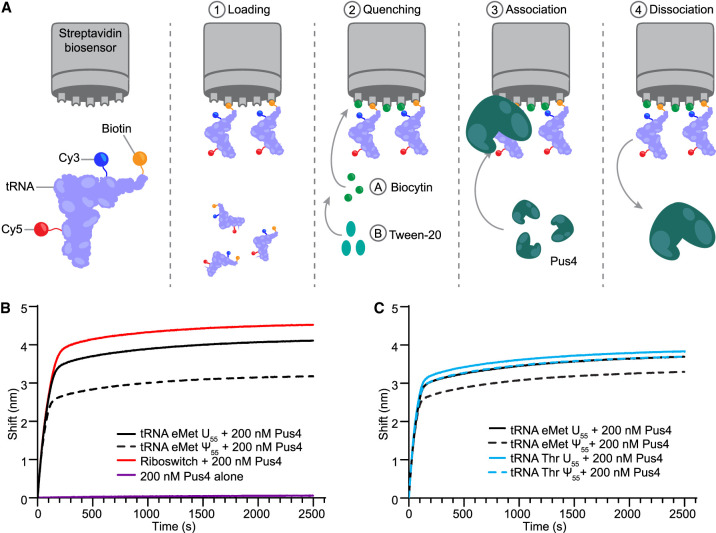
Pus4 binding to tRNA measured using biolayer interferometry. (*A*) Summary of BLI experimental design. Biotinylated and Cy3- and Cy5-labeled tRNAs are immobilized onto streptavidin-coated probes (1) that then undergo two quenching steps and baselining (2). The probe is then transferred into buffer with purified Pus4, where association of Pus4 with immobilized tRNAs occurs (3). The probe is then transferred to a buffer without protein, permitting dissociation (4). (*B*) BLI traces showing the association phase of 200 nM Pus4 to U_55_ (black solid) and Ψ_55_ (black dashed) tRNA eMet, to an unrelated RNA (riboswitch; red), and to a passivated probe lacking RNA (purple). Traces are aligned to start at a shift of 0 nm. (*C*) Same as *B*, but with U_55_ (black solid) and Ψ_55_ (black dashed) tRNA eMet and U_55_ (blue solid) and Ψ_55_ (blue dashed) tRNA Thr. Complete traces in Supplemental Figure S2.

**FIGURE 4.**
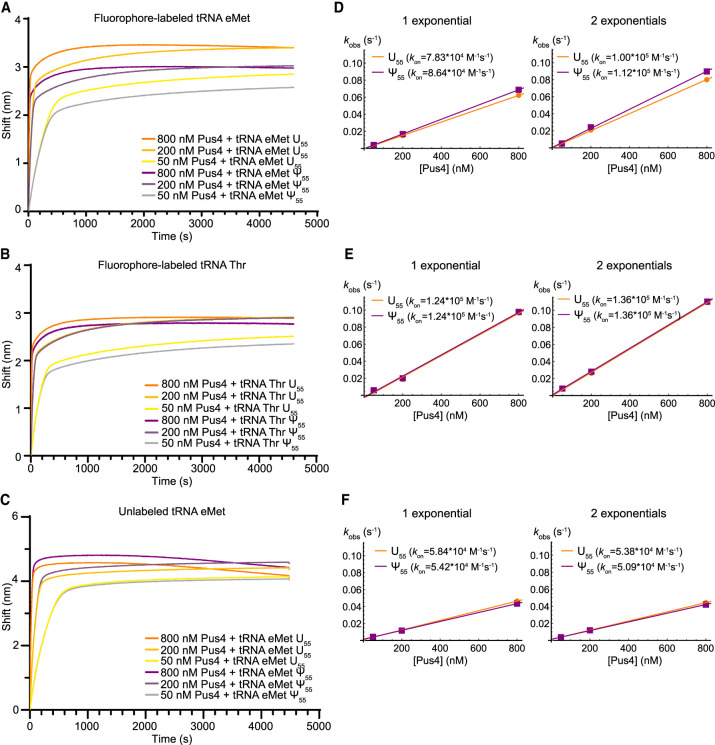
Pus4 binds tRNAs with a high *k*_on_, but a low *k*_off_, with similar affinities for unmodified and premodified tRNAs. (*A–C*) BLI traces showing the association phase of 50, 200, and 800 nM Pus4 to various tRNAs. Traces are aligned to start at a shift of 0 nm. (*A*) Fluorophore-labeled U_55_ (orange) and Ψ_55_ (purple) tRNA eMet. (*B*) Fluorophore-labeled U_55_ (orange) and Ψ_55_ (purple) tRNA Thr. (*C*) Unlabeled U_55_ (orange) and Ψ_55_ (purple) tRNA eMet. (*D–F*) Linear fits to the observed association rate at three Pus4 concentrations. The slope of the fit, representing *k*_on_, is indicated for each plot. (*Left* columns) *k*_obs_ determined from a mono-exponential fit to the time range of the association phase during which ∼75% of the BLI shift occurs. (*Right* columns) *k*_obs_ determined from a biexponential fit to the first 1000 sec of the association phase for unlabeled tRNA eMet and the entire association phase for the others. (*D*) Fluorophore-labeled U_55_ (orange) and Ψ_55_ (purple) tRNA eMet. (*E*) Fluorophore-labeled U_55_ (orange) and Ψ_55_ (purple) tRNA Thr. (*F*) Unlabeled U_55_ (orange) and Ψ_55_ (purple) tRNA eMet.

We utilized BLI to assess the binding specificity of Pus4 and the timescales of its association with and dissociation from both substrate and nonsubstrate RNAs. RNAs were immobilized on a streptavidin-coated probe that was then passivated to prevent nonspecific adsorption of protein, then exposed to buffer containing Pus4 protein (the “association phase”), and finally exposed to buffer lacking protein (the “dissociation phase”) (Fig. 3A; Supplemental Fig. S2A). A BLI “shift” is observed when material accumulates on the probe, such as protein binding to immobilized tRNA during the association phase. At a concentration of 200 nM protein, the association curves show rapid binding of Pus4 to tRNA eMet and tRNA Thr, with the accumulation rate slowing after ∼100 sec (Fig. 3B,C; Supplemental Fig. S2B,C; replicate data in Supplemental Fig. S3). While accumulation plateaued after ∼15 min when Pus4 was present at our highest tested concentration (800 nM), it failed to plateau even after 75 min for Pus4 at 50 or 200 nM concentrations (Fig. 4). The BLI shift at 200 nM Pus4 eventually converged with the data for 800 nM, suggesting that complete binding occupancy was achieved in this concentration range. We confirmed this using nondenaturing gel electrophoresis as an orthogonal tRNA binding assay, which showed that in free solution, all tRNA eMet molecules were bound after 30 min incubation with Pus4 at concentrations of 200 nM or higher (Supplemental Fig. S4). Combined with the prior observation that both tRNA eMet and tRNA Thr were robustly modified within 2 min during the in vitro modification experiment (in which Pus4 was present at 1 µM) (Fig. 2), we presumed that the majority of the immobilized tRNAs were pseudouridylated in the early period of this association phase once engaged with Pus4. The in vitro modification experiment and BLI experiments were performed in the same buffer (with the exception of Tween-20 used to suppress nonspecific binding in BLI), at the same temperature, and using the same molar ratio of protein to tRNA (3.33), lending support to this assumption.

We analyzed the association of Pus4 to tRNA quantitatively by modeling the initial binding of Pus4 as a reversible process occurring under pseudo-first order conditions, which predicts that Pus4 should accumulate on the tRNA with a profile described by an exponential function (details in Materials and Methods). To obtain accurate *k*_on_ values, we devised and compared multiple fitting approaches to isolate the kinetics of initial binding from the kinetics of the continued slow accumulation observed by BLI and the catalysis observed by DRS, which could cause the binding kinetics to change over time (Supplemental Fig. S5). In one approach, we isolated this phase of association by performing mono-exponential fits (as predicted from pseudo-first order kinetics) to the time period over which the first ∼75% of the overall BLI shift occurs. In the second approach, we modeled both the initial binding and continued slow accumulation by fitting the first 1000 sec of the association phase with a biexponential function and took the faster component to represent initial binding to the preexisting distribution of tRNA structures. Both approaches revealed that the on-rate (*k*_on_) was slightly faster for tRNA Thr (1.36 × 10^5^ m^−1^ sec^−1^ for U_55_ from the biexponential fit) than for tRNA eMet (1.00 × 10^5^ m^−1^ sec^−1^) (Fig. 4D,E). These on-rates are comparable to those previously observed for yeast Pus1 binding to tRNA^Phe^ and tRNA^Val^ (4–6 × 10^5^ m^−1^ sec^−1^) (Arluison et al. 1999). These fits could in principle have been used to obtain *k*_off_; however, the off-rate during the initial phase of Pus4 accumulation was indistinguishable from zero. When the BLI probe was transferred into a solution lacking protein, an initial fast phase of dissociation was observed, but even after 75 min, <10% of the shift observed in the association phase was reversed (Supplemental Fig. S2B,C). This suggests that Pus4 binds all RNAs investigated with high affinity and dissociates very slowly.

To test whether the fluorophores altered Pus4 binding affinity to tRNAs, we prepared a synthetic tRNA eMet with the same sequence as above, but without Cy3 and Cy5 fluorophores (Fig. 4C; Supplemental Fig. S6). The BLI curves showed the same overall shape with a somewhat lower *k*_on_ value (5.38 × 10^4^ m^−1^ sec^−1^) than observed for labeled tRNA eMet, an impact comparable in size to the variation between different tRNAs (labeled tRNA eMet vs. labeled tRNA Thr). We conclude that while the labels exert a small effect on Pus4 binding, in general our strategy for investigating the conformational ensemble of tRNA using smFRET is not confounded by the effects of these fluorophores on the binding behavior of Pus4.

After the initial binding phase that was used for kinetic analysis, we noted other features of Pus4 binding behavior. In the highest concentration tested (800 nM protein), we observed that after the binding plateaued, a mild dissociation occurred slowly over the remainder of the measurement (Fig. 4). The effect was more distinct in the unlabeled tRNAs in comparison to the fluorophore-labeled, but was present in all tRNAs. This appeared to be a distinct phase from the binding phase; thus we did not incorporate it into our kinetic analysis. Secondly, the slow, steadily increasing second phase of the association curve observed at all concentrations of Pus4 tested may represent (1) gradual “trapping” of tRNA or Pus4 molecules that began the association phase in conformations not amenable to binding or (2) Pus4 multimerization such that the stoichiometry of Pus4:tRNA exceeded 1:1 on some of the immobilized tRNA molecules. Multimerization is supported by the shift of Pus4:tRNA bands to the wells for the highest Pus4 concentrations (Supplemental Fig. S4) and might be facilitated by the GST tag that is known to dimerize (Fabrini et al. 2009). To measure the extent to which Pus4 multimerization could impact tRNA binding, we incubated total tRNA isolated from yeast cells with recombinant Pus4 and performed sucrose gradient ultracentrifugation to separate protein:RNA complexes (Supplemental Fig. S7). If multimerization of Pus4 under these conditions reduced tRNA binding, we would expect no evident shift in the distribution of protein complexes upon addition of tRNA. However, addition of tRNA caused a global shift of Pus4 to fractions in the gradient containing a higher percentage of sucrose, forming complexes with an apparent molecular weight of ∼200 kDa (implying complexes of two GST-Pus4 with two tRNAs). Additionally, our smFRET measurements (described below) were performed on sparsely surface-immobilized tRNAs, in which the average distance between tRNA molecules greatly exceeds the size of a protein dimer or even multimer. This would preclude the formation of complexes containing more than one tRNA molecule. In summary, while we can detect Pus4 multimers in our experimental conditions, we do not believe that they represent the dominant form of Pus4 that is interacting with immobilized tRNAs that are the principal substrates that we measured, and moreover, our Pus4 samples demonstrated robust catalysis (Fig. 2).

### Pus4 also binds robustly to premodified tRNA and nonsubstrate RNA

To test whether Ψ_55_ altered the binding of Pus4 to tRNA, we tested synthetic tRNAs that had pseudouridine present at position 55 using BLI. Although the BLI curve of Ψ_55_ tRNA eMet exhibited a smaller maximum shift than that of U_55_ (Fig. 4A), nondenaturing gel electrophoresis indicated that this still corresponded to complete binding occupancy at Pus4 concentrations at or above 200 nM (Supplemental Fig. S4). Furthermore, the overall association and dissociation profile was similar between the unmodified and premodified variants of both tRNAs, and *k*_on_ values were unchanged or slightly higher with Ψ_55_ tRNA (1.12 × 10^5^ m^−1^ sec^−1^ for Ψ_55_ tRNA eMet and 1.36 × 10^5^ m^−1^ sec^−1^ for Ψ_55_ tRNA Thr), indicating weak discrimination between binding substrate and product. We also performed experiments with an RNA that is not a natural substrate of Pus4 but is similarly sized to tRNA: the bacterial class II NAD^+^ riboswitch, a 61 nt RNA that adopts a pseudoknot structural motif to regulate translation initiation (Panchapakesan et al. 2021; Conoan Nieves and Widom 2024). As we observed for tRNA, Pus4 rapidly associated with the riboswitch, followed by slow continued accumulation and slow dissociation (Fig. 3B, Supplemental Fig. S2B). It is worth noting that previous studies have shown that pseudouridine synthases can be promiscuous binders of RNA (Behm-Ansmant et al. 2003; Carlile et al. 2019; Purchal et al. 2022; Grünberg et al. 2023), consistent with their roles of modifying multiple sequences and structures in various classes and sizes of RNA. For example, Pus1 has been found to have broad affinity for RNA, even those lacking defining tRNA structural features, as well as tight binding to catalytic products (e.g., *K*_D_ = 14 nM for pseudouridylated tRNA^Val^) (Arluison et al. 1999). In addition to tRNA, Pus4 modifies mRNA (Carlile et al. 2014; Lovejoy et al. 2014; Schwartz et al. 2014) and mt-rRNA (Begik et al. 2021), in vivo. Our data suggest that, like Pus1, Pus4 also exhibits promiscuous binding. This may promote Pus4's ability to facilitate RNA remodeling more generally, even if its primary catalytic substrate is tRNA.

### tRNA undergoes dynamic fluctuations between three predominant conformational states

We utilized smFRET to investigate how tRNA conformations are affected by Pus4 binding and pseudouridylation. The biotinylated- and fluorophore-labeled tRNA constructs used for BLI were immobilized on microscope slides, and fluorescence from single tRNA molecules was recorded using wide-field prism-based total internal reflection fluorescence (TIRF) microscopy. We recorded short movies (∼2 min) sequentially across different regions of the slide of tRNA alone, as well as tRNA during up to 30 min of incubation with 200 nM Pus4 protein. Cy3 and Cy5 fluorescence intensity traces were extracted from individual molecules, and data were combined to achieve ∼200 traces for each condition. To observe the evolution of the conformational landscape over tens of minutes, the data were combined into discrete time bins of 2–6, 7–9, 10–15, and 30–34 min after introduction of the imaging buffer, either with or without Pus4. FRET efficiency (*E*_FRET_) histograms were compiled from the first 50 frames of all traces within each condition.

To accurately recover signals reflecting the distance between the donor and acceptor fluorophores, and therefore the structure of the tRNA, FRET traces were corrected for (1) cross talk between detection channels and (2) gamma (γ) factor, which quantifies the relative detection efficiencies and fluorescence quantum yields of Cy3 and Cy5 (Supplemental Fig. S8). We determined the γ factor utilizing changes in donor and acceptor intensity upon acceptor photobleaching (McCann et al. 2010), which yielded a distribution with a median value of 2.4 in the absence of Pus4 and 3.0 in the presence of Pus4 (Supplemental Fig. S8C,D). Given the significant adjustment to the raw data imposed by a γ factor so far from 1 (Supplemental Fig. S8A), we also tested whether utilizing a molecule-by-molecule γ factor correction would improve the resolution of different FRET states. However, restricting the analysis to molecules for which individual γ values could be calculated (molecules in which the acceptor photobleached first) skewed the histogram without sharpening its features (Supplemental Fig. S8E–G). We thus elected to utilize the median of the distribution (with or without Pus4 as appropriate) as a global γ correction factor, permitting inclusion of all traces.

**FIGURE 5.**
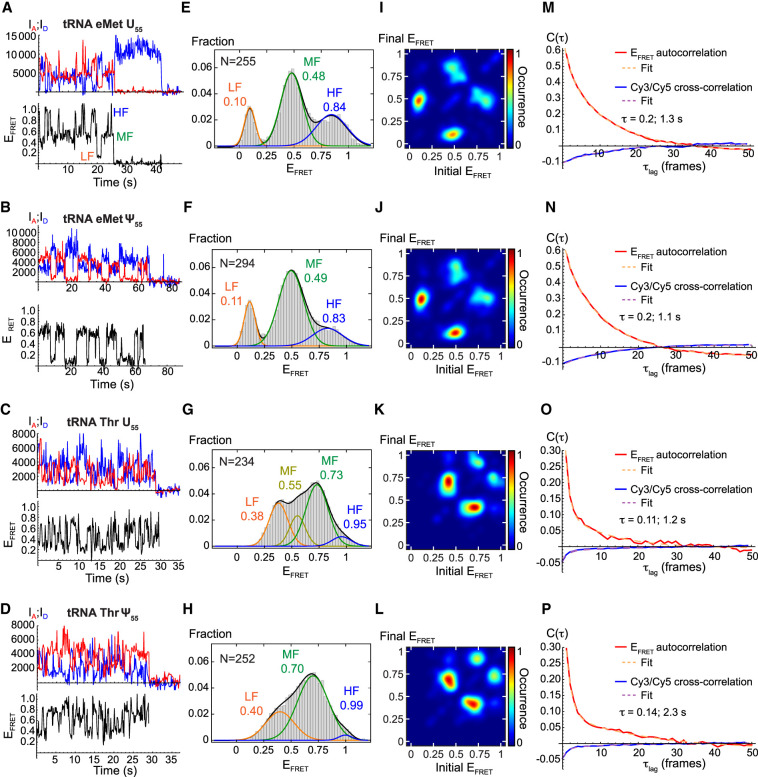
smFRET data on tRNAs in the absence of Pus4. (*A*–*D*) Representative smFRET traces of (*A*) U_55_ tRNA eMet, (*B*) Ψ_55_ tRNA eMet, (*C*) U_55_ tRNA Thr, (*D*) Ψ_55_ tRNA Thr. (*Top*) Donor (blue) and acceptor (red) intensities. (*Bottom*) FRET efficiency. Occurrences of the LF, MF, and HF states are indicated in *A*. For clarity, the *E*_FRET_ traces are truncated after donor photobleaching. Only trace segments prior to photobleaching of either dye were subjected to further analysis. (*E*–*H*) *E*_FRET_ histograms compiled from the first 50 frames of the number of traces indicated by “N” for (*E*) U_55_ tRNA eMet, (*F*) Ψ_55_ tRNA eMet, (*G*) U_55_ tRNA Thr, (*H*) Ψ_55_ tRNA Thr. Three- or four-Gaussian fits are overlaid with the peak centers labeled. (*I*–*L*) TODPs for (*I*) U_55_ tRNA eMet, (*J*) Ψ_55_ tRNA eMet, (*K*) U_55_ tRNA Thr, (*L*) Ψ_55_ tRNA Thr. The intensity of the heat map at position (*X*,*Y*) quantifies the fraction of molecules that transition from *E*_FRET_ = *X* to *E*_FRET_ = *Y* at least once during the measurement. (*M*–*P*) FRET autocorrelation (red) and Cy3/Cy5 cross-correlation (blue) functions for (*M*) U_55_ tRNA eMet, (*N*) Ψ_55_ tRNA eMet, (*O*) U_55_ tRNA Thr, (*P*) Ψ_55_ tRNA Thr. Global biexponential fits with the same time constants for autocorrelation and cross-correlation functions are overlaid in dashed lines, and the two time constants are indicated.

Inspection of the corrected traces revealed that in the absence of protein, all four tRNA variants investigated—U_55_ and Ψ_55_ tRNA eMet and U_55_ and Ψ_55_ tRNA Thr—dynamically sampled at least three distinct conformational states (Fig. 5A–D). The shapes of the distributions and FRET efficiencies, however, differed between the eMet and Thr tRNA sequences. In the histograms of U_55_ and Ψ_55_ tRNA eMet, which were well-fit with three Gaussian distributions, a low-FRET efficiency feature (LF)—representing a conformation in which the fluorophores are most separated from each other relative to other states—is centered around *E*_FRET_ = 0.1. An LF feature could arise from molecules that are either less structured or that adopt a structure such as an extended helix that enforces separation of the fluorophores. Mid-FRET (MF) and high-FRET (HF) features—representing conformations in which the fluorophores are closer to each other relative to LF—were observed for both U_55_ and Ψ_55_ tRNA eMet and centered around *E*_FRET_ = 0.48 and 0.83, respectively (Fig. 5E,F). The HF state was moderately more populated in U_55_ than in Ψ_55_ tRNA eMet. LF, MF, and HF states were previously observed in smFRET analysis of the human mt-tRNA^Lys^ and were assigned to unfolded (U), extended hairpin (E), and cloverleaf-based L-shape (C) conformations, respectively (Dammertz et al. 2011). The histograms of U_55_ and Ψ_55_ tRNA Thr exhibited broad, asymmetric distributions centered around *E*_FRET_ = 0.6. These were well-fit with either four (U_55_) or three (Ψ_55_) Gaussian distributions (Fig. 5G,H) with centers ranging from *E*_FRET_ = 0.38 to 0.99, consistent with the variety of states observed in the traces (Fig. 5C,D).

We analyzed the dynamics of the four tRNAs by performing hidden Markov modeling (HMM) (Blanco and Walter 2010). For tRNA eMet, we used a four-state model (Supplemental Fig. S9A) that included the LF, MF, and HF states, in addition to a second HF state (HF2) centered at *E*_FRET_ = 0.95, which was observed in a fraction of molecules too small to yield a distinct feature in the histogram (5% in U_55_ and 2% in Ψ_55_). The model allowed for transitions between all pairs of the LF, MF, and HF states, with the HF2 state occupied only statically, in accordance with the small number of traces that exhibited it. The isolated HF2 state was omitted for tRNA Thr based on inspection of traces, and the *E*_FRET_ values of the LF, MF, and HF states were adjusted to match those obtained from three-Gaussian fits to their histograms (Supplemental Fig. S9B). The pathways of transitions among these states were visualized using transition occupancy density plots (TODPs), which show the prevalence of dynamic or static behavior across hundreds of molecules within each condition (Fig. 5I–L). A feature on the diagonal axis of a TODP (i.e., lower left corner to upper right corner) indicates a population of static molecules that remain in one state throughout their measurement, while an off-diagonal feature at position (*X*,*Y*) indicates a population of dynamic molecules that transition from states with *E*_FRET_ = *X* (initial) to *E*_FRET_ = *Y* (final) at least once during the measurement. This analysis showed that the majority of molecules (>80% for U_55_ and Ψ_55_ variants of both tRNAs) exhibited dynamic behavior involving fluctuations between conformations. This is consistent with previous bulk (Cole et al. 1972; Bhaskaran et al. 2014) and smFRET (Voigts-Hoffmann et al. 2007) studies that showed that tRNAs can interconvert between multiple secondary and tertiary structures at physiological salt and ambient temperature. In U_55_ and Ψ_55_ variants of both tRNAs, bright off-diagonal features in the TODPs indicate that molecules readily and reversibly interconverted between the LF and MF states and between the MF and HF states (Fig. 5I–L). Significantly, almost no intensity was observed at the locations corresponding to transitions between the LF and HF states in any of the four tRNAs investigated, indicating that interconversion between these two states requires a molecule to transition through an intermediate MF state.

For U_55_ and Ψ_55_ tRNA eMet, HMM analysis further revealed transition rates of 0.3–0.8 sec^−1^ between LF and MF states and slightly faster transition with rates of 0.8–1.5 sec^−1^ between MF and HF states (Table 1; Supplemental Fig. S10). The overall rates of conformational fluctuations in each tRNA were also quantified in a model-free manner using cross-correlation and autocorrelation analyses (details in Materials and Methods), which identify how rapidly Cy3 and Cy5 intensities become uncorrelated with one another within a collection of smFRET traces. Consistent with the HMM results, the correlation analysis confirmed that fluctuations in FRET efficiency occurred on a timescale of ∼1 sec in U_55_ and Ψ_55_ tRNA eMet (Fig. 5m,N). For U_55_ and Ψ_55_ tRNA Thr, fluctuations were notably faster, with HMM-derived transition rates between the LF and MF states almost four times faster than observed for tRNA eMet (Table 1), and biphasic correlation functions dominated by fluctuations on a timescale <0.15 sec rather than the ∼1 sec observed for tRNA eMet (Fig. 5O,P). This approaches the 100 msec time resolution of our smFRET measurements, suggesting that the indistinct features of the histograms of tRNA Thr may have arisen in part from time averaging across transitions between states. In summary, our analysis revealed that U_55_ and Ψ_55_ tRNA eMet and tRNA Thr all undergo frequent conformational changes involving substantial adjustments in their three-dimensional architecture, with their MF states acting as near-obligate intermediates between LF and HF states.

**TABLE 1.** HMM-derived rate constants (weighted average from a biexponential fit) for transitions between each pair of interconverting FRET states

tRNA	*k*_LF→MF_ (sec^−1^)	*k*_MF→LF_ (sec^−1^)	*k*_MF→HF_ (sec^−1^)	*k*_HF→MF_ (sec^−1^)
U_55_ tRNA eMet	0.58	0.34	1.00	1.43
Ψ_55_ tRNA eMet	0.79	0.58	0.79	1.49
U_55_ tRNA Thr	2.83	1.26	0.90	1.59
Ψ_55_ tRNA Thr	2.64	1.22	0.91	1.70

Fits shown in Supplemental Figure S10.

### Pus4 alters the conformational states and transition dynamics of tRNA eMet

**FIGURE 6.**
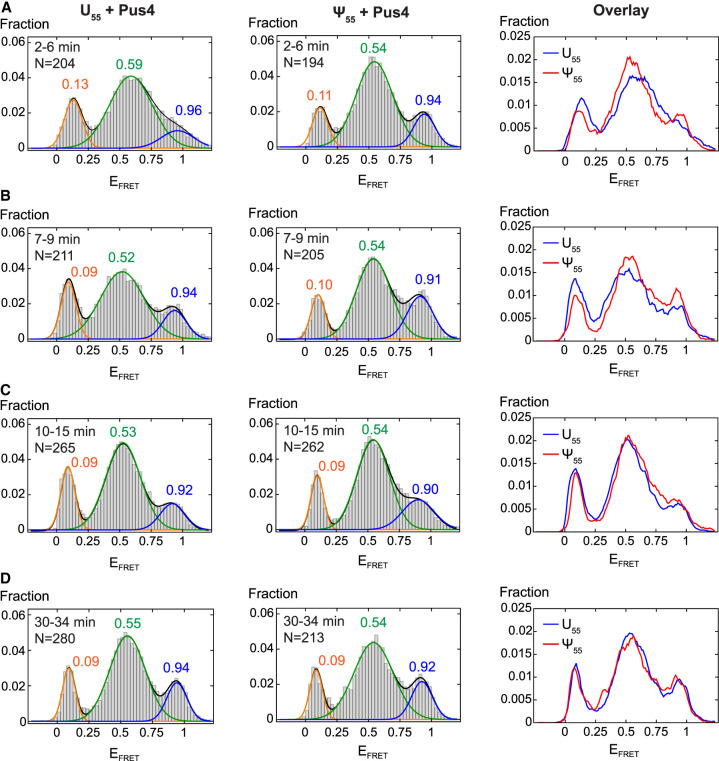
smFRET histograms for U_55_ and Ψ_55_ tRNA eMet in the presence of 200 nM Pus4. (*A*) Histograms compiled from movies recorded 2–6 min after injection of Pus4. (*Left* column) U_55_ + Pus4 histogram with three-Gaussian fit overlaid. The *E*_*FRET*_ value of each state is indicated. (*Middle* column) Ψ_55_ + Pus4 histogram with three-Gaussian fit overlaid. (*Right* column) Overlay of U_55_ and Ψ_55_ histograms. (*B*) Same as *A* but compiled from movies recorded 7–9 min after injection of Pus4. (*C*) Same as (*A*,*B*) but compiled from movies recorded 10–15 min after injection of Pus4. (*D*) Same as (*A*–*C*) but compiled from movies recorded 30–34 min after injection of Pus4.

**FIGURE 7.**
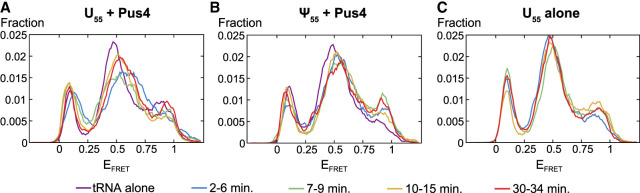
Overlaid smFRET histograms throughout incubation of (*A*) U_55_ tRNA eMet with 200 nM Pus4, (*B*) Ψ_55_ tRNA eMet with 200 nM Pus4, (*C*) U_55_ tRNA eMet with imaging buffer lacking Pus4. (Blue) Histogram compiled from traces recorded 2–6 min after introduction of imaging buffer containing or lacking Pus4; (green) 7–9 min; (yellow) 10–15 min; (red) 30–34 min. (*A*,*B*) Additionally show the histograms of the corresponding tRNA alone after 10–15 min of incubation with imaging buffer lacking Pus4 (purple).

**FIGURE 8.**
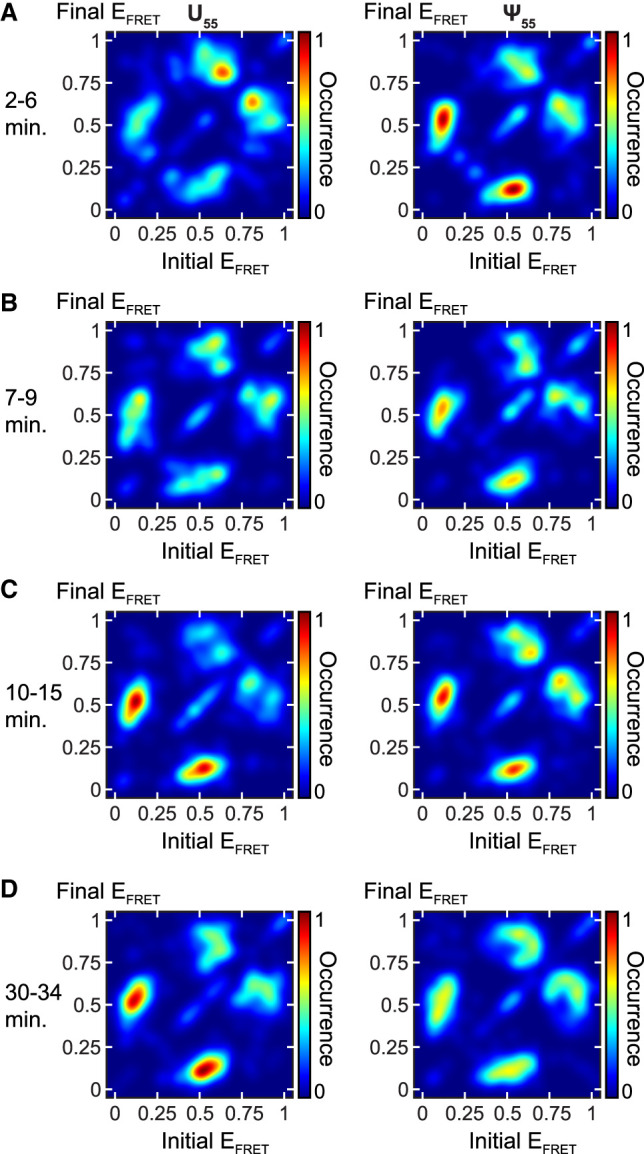
TODPs for U_55_ (*left* column) and Ψ_55_ (*right* column) tRNA eMet in the presence of 200 nM Pus4 compiled from traces recorded (*A*) 2–6 min after injection of protein, (*B*) 7–9 min, (*C*) 10–15 min, (*D*) 30–34 min.

After Pus4 was introduced into a sample chamber containing immobilized U_55_ or Ψ_55_ tRNA eMet, a rapid increase in brightness of both Cy3 and Cy5 indicated that the protein was binding to the RNA (Supplemental Table S2), a phenomenon known as “protein-induced fluorescence enhancement” (PIFE) (Hwang et al. 2011). This increase was largely complete by the earliest time window that we probed by smFRET, 2–6 min, which begins when the initial fast phase of Pus4 association observed by BLI is nearly complete and continues into the slow accumulation phase (Fig. 3B,C). Concurrently, an increase in conformational complexity was observed, indicating that the protein changed the free energy landscape of tRNA molecules. Two to 6 min after addition of protein, the histogram of U_55_ tRNA broadens, and all three features shift to higher *E*_FRET_ (Figs. 6A, 7A), potentially indicating changes to specific tertiary contacts previously held. Inspection of traces suggested that the observed increase in complexity originates from interconversion between mid-FRET “microstates” with *E*_FRET_ ∼0.4 and 0.7 (Supplemental Fig. S11A). Although we used only one MF state in the HMM model to avoid overfitting and facilitate comparison to data recorded in the absence of protein (Supplemental Fig. S9A), the presence of additional microstates is reflected in TODPs (Fig. 8A–D). Specifically, the features present in tRNA alone became broader in the presence of Pus4, and transitions were observed between states with a greater variety of *E*_FRET_ values (Fig. 5I,J vs. Fig. 8A). We hypothesize that these microstates involve remodeling of the T-loop in order to facilitate pseudouridylation, as observed in crystallography data of the bacterial homolog TruB with tRNA (Hoang and Ferré-D'Amaré 2001; Pan et al. 2003). The shift to higher *E*_FRET_ in the histogram is partially reversed in the 7–9 min time window (Fig. 6). Given that BLI (Fig. 3) and PIFE (Supplemental Table S2) both indicate that protein occupancy increases monotonically throughout this time period, the nonmonotonic changes in the peak *E*_FRET_ values and feature widths suggest that the stably bound protein–RNA complex evolves in structure after its initial assembly. By 30–34 min after introduction of Pus4 (at which point our gel shift assay indicated that all tRNA molecules were bound by Pus4 in solution; Supplemental Fig. S4), the conformational ensemble resolves back into distinct LF, MF, and HF features, with MF and HF centered at higher *E*_FRET_ than before exposure to Pus4 (Fig. 7A). The TODPs reflect this as well, showing a convergence of features by the 30–34 min time window (Fig. 8D). While our approach precluded real-time, simultaneous measurement of pseudouridylation of the immobilized tRNA, our in vitro pseudouridylation assay showed that the majority of tRNAs were pseudouridylated by Pus4 within 2 min (Fig. 2). Therefore, results over the course of these smFRET experiments reflect the enduring influence of Pus4 binding and catalysis on the trajectory of the tRNA conformations sampled.

As a control, we binned smFRET data recorded on U_55_ tRNA in the absence of Pus4 into the same time windows (Fig. 7C; Supplemental Fig. S12). We observed small changes in the relative amplitudes of the histogram features, particularly LF and HF. These changes were likely due to the finite number of molecules analyzed for each time window and the gradual activation and depletion of the oxygen scavenging system used to prolong fluorophore activity. Importantly, however, U_55_ tRNA alone did not exhibit the curve broadening, shifts in *E*_FRET_, or increase in overall intensity (Supplemental Table S2) that had characterized its time evolution in the presence of Pus4.

To help isolate the influence of Pus4 binding and remodeling of tRNA from its pseudouridylation activity, we performed smFRET experiments with the premodified Ψ_55_ tRNA eMet in the presence of Pus4. While MF microstates were observed in Ψ_55_ tRNA eMet at early times (Supplemental Fig. S11B), as we also observed for U_55_ tRNA eMet, Ψ_55_ tRNA histograms and TODPs exhibited less dramatic changes. Specifically, in the 2–6 min window following injection of Pus4, Ψ_55_ tRNA exhibited a smaller shift of the MF state toward higher *E*_FRET_ than U_55_ and an increase in the population of the HF state (Fig. 6A). Ψ_55_ tRNA retained the same shape in its histogram throughout the remainder of the time course, exhibiting only minor fluctuations of the relative populations of its states (Figs. 6, 7B). These fluctuations were of comparable size to those observed in tRNA alone (Fig. 7C; Supplemental Fig. S12), so we conclude that the impacts of Pus4 on the conformational ensemble of Ψ_55_ tRNA are largely complete after 2–6 min of interaction. This stands in contrast to U_55_ tRNA, which continues to evolve through at least 15 min after injection of Pus4 despite the protein binding at a similar rate and remaining bound to both throughout the time course (as indicated by BLI and PIFE) and Pus4 presumably pseudouridylating most U_55_ tRNA molecules within 2 min (Fig. 2). By 10–15 min of incubation with Pus4, U_55_ tRNA exhibited only a small skew toward lower *E*_FRET_ relative to Ψ_55_, and their histograms were nearly superimposable by 30–34 min (Fig. 6C,D). We hypothesize that Ψ_55_ tRNA thus bypassed some of the conformational changes that U_55_ tRNA transitioned through during its catalysis and subsequent remodeling by Pus4.

## DISCUSSION

In the decades since the three-dimensional structure of tRNA was elucidated (Kim et al. 1974), it has been presented as an archetype of structured noncoding RNA. Yet every tRNA undergoes many processing steps during biogenesis—including trimming, tailing, and the addition of multiple chemical modifications—necessitating many conformational changes. In this study we combined biophysical and biochemical approaches to document the dynamics of conformational changes that two yeast tRNAs can undergo over time. We then measured how this chronology of tRNA structural dynamics changed upon binding by a ubiquitous pseudouridine synthase that modifies many tRNAs in nature. These data demonstrated how Pus4 impacts tRNA conformation through its binding and catalysis, leading to a more well-defined ensemble of conformations when the RNA–protein complex reached equilibrium. This outcome was reached after a period of minutes if catalysis was bypassed (i.e., Pus4 incubated with a premodified tRNA), rather than tens of minutes if catalysis occurred.

We find that tRNA eMet and tRNA Thr each sample complex free energy landscapes, with at least three conformational states resolved by smFRET. Both exhibit a hierarchical folding landscape, with their MF states as intermediates, though tRNA Thr exhibits faster dynamics. Furthermore, tRNA eMet maintains a dynamic conformational landscape of LF, MF, and HF states throughout its interactions with Pus4 and independently of its modification status, with additional MF states populated upon engagement with Pus4. In the absence of protein, the *E*_FRET_ values of these features align reasonably closely with FRET states previously observed in human mt-tRNA^Lys^ under equivalent ionic conditions of 50 mM buffer, 100 mM monovalent cation, and no divalent cation (Dammertz et al., 2011). However, we consistently observe a larger HF population in the yeast tRNA eMet than previously observed in mt-tRNA^Lys^, which we propose can be reconciled by predicted free energies. The cloverleaf secondary structure of tRNA eMet has a predicted Δ*G*_folding_ = −18.9 kcal/mol (details of free energy calculations in Materials and Methods), significantly more stable than −16.1 kcal/mol for mt-tRNA^Lys^ studied in Voigts-Hoffmann et al. (2007) and subsequent studies (Supplemental Fig. S13A,E; Kobitski et al. 2008, 2011; Dammertz et al. 2011).

mt-tRNA^Lys^ also substantially populated an MF state with *E*_FRET_ ∼0.4 that interconverted with C on a timescale of hundreds of milliseconds (Voigts-Hoffmann et al. 2007). This state was assigned to an extended hairpin secondary structure (E) that had previously been identified in this tRNA by biochemical methods (Helm et al. 1998). Interconversion between C- and E-type structures was also observed using biochemical methods in a different tRNA, mt-tRNA^Leu^ (Bhaskaran et al. 2014). Consistent with these experimental observations, the E structure of mt-tRNA^Lys^ is predicted to have a free energy of −15.3 kcal/mol, within 1 kcal/mol to that of the C structure (Supplemental Fig. S13E,F). To place our results into this context, we constructed a plausible E-like secondary structure of yeast tRNA eMet by identifying potential base pairs that could be formed between nucleotides in the T- and D-loops and adjacent stems (Supplemental Fig. S13B). Unlike mt-tRNA^Lys^, however, at Δ*G* = −12.9 kcal/mol, this E-like structure of tRNA eMet is predicted to be 6 kcal/mol less stable than its C structure (Supplemental Fig. S13A,B). Likewise, tRNA Thr is predicted to have a C structure that is 7 kcal/mol more stable than a plausible E structure (−24.4 and −17.4 kcal/mol, respectively) (Supplemental Fig. S13C,D). In light of the substantial free energy preference for the C secondary structures of tRNA eMet and tRNA Thr, we consider it unlikely that the dominant MF states that we observe both with and without Pus4 arise from E-like secondary structures. Instead, the MF states observed in tRNA alone and the variations thereof that are populated in tRNA eMet in the presence of Pus4 may be explained by (1) relatively minor changes in secondary structure such as fraying/unpairing of the acceptor stem that contains the donor fluorophore and/or (2) changes in tertiary structure such as breaking of the T-loop–D-loop interaction. These structures have been observed in *E. coli* tRNAs in vivo under stress conditions (Yamagami et al. 2022). Prior steady-state and kinetic measurements also identified multiple interconvertible tertiary structures under conditions generally favoring the C secondary structure (Cole and Crothers 1972).

While our FRET approach reveals tRNA dynamics from the perspective of the distance between the anticodon stem and acceptor stem, complementary methods have provided insight into other degrees of freedom that characterize the accessible structural states. A recent study that used single particle cryo-EM and melting temperature assays found that tRNA folding was strongly influenced by the tertiary structure–stabilizing interaction between the D- and T-loops, and that this interaction itself was generally promoted by Ψ_55_ (Biela et al. 2025). Another study performed NMR on *E. coli* tRNA^fMet^ and found that Ψ_55_ and neighboring modifications have pronounced effects on local structural dynamics (Biedenbänder et al. 2022). These dynamics were shown to facilitate interactions between the T-loop and D-loop and improve the overall stability of the tertiary fold of the tRNA. However, modification did not induce dramatic shifts in global structure, in agreement with our results showing largely similar global conformational ensembles for tRNA with and without Ψ_55_.

Future studies of additional tRNA and Pus4 variants may improve delineation of the structures that give rise to the observed FRET states and their functional significance. For example, alternate placement of FRET pairs would permit degrees of freedom other than the anticodon stem–acceptor stem distance to be probed. Placing a third fluorophore in the D-loop of the tRNA—so as not to interfere with the T-loop pseudouridylation but enable detection of altered T-loop/D-loop interactions—may yield a more complete picture of the three-dimensional architecture of the tRNA as it is remodeled. Alternatively, three-color measurements involving covalent attachment of a fluorophore to Pus4 protein, with the remaining two fluorophores on tRNA, may simultaneously permit detection of Pus4 binding and tRNA structural remodeling over time by more clearly differentiating protein-bound from unbound conformations. A method to measure pseudouridylation in real-time on the microscope slide would also permit direct correlation of activity to structural remodeling, or the kinetics of binding if measured on a BLI probe. The assays developed in this study could also be applied to a catalytically impaired mutant of Pus4, such as the previously reported R286K (Shaw et al. 2024). This could further define the functional significance of the observed tRNA conformations, for example, in determining if a lack of pseudouridylation activity changes the kinetics of remodeling of the T-loop by bound Pus4.

Our work herein began with the goal to characterize the conformational dynamics of tRNA as influenced by pseudouridylation and interaction with a pseudouridine synthase. Guided by our results, we propose that each instance of Pus4 engaging with tRNA follows a three-to-four-step process: (1) Initial binding of Pus4 occurs, (2) Pus4 “tests” whether Ψ_55_ is already present, (3) if Ψ_55_ is absent, Pus4 may convert U_55_ to Ψ_55_, (4) Pus4 remodels tRNA into a new distribution of structures. Pus4 effectively discriminates U from Ψ, remodeling premodified tRNA toward the final structural ensemble within minutes. Upon initial engagement with unmodified or premodified tRNA, Pus4 induces an additional population of intermediate (MF) states. Such structural states may play a role in discrimination between substrate and product.

Overall, our results demonstrate two important layers of influence on tRNA conformational dynamics: (1) chemical catalysis and (2) remodeling resulting from extended and recurrent interactions with modification enzymes. Why Pus4 would promote such malleability in tRNA conformational dynamics on already pseudouridylated tRNAs remains an important question. Since Pus4 catalysis can strongly influence the addition of subsequent modifications in vivo (Shaw et al. 2024), and some are added over many hours after pseudouridylation (Barraud et al. 2019), we propose that part of the role of Pus4 is to make tRNA structure amenable to binding and modification by subsequent modification enzymes that are critical for tRNA function.

## MATERIALS AND METHODS

### Ligation of synthetic tRNA sequences

Full-length tRNA constructs were made by ligation of two RNA oligonucleotides (Dharmacon, HPLC-purified by the manufacturer) using a DNA splint (Integrated DNA Technologies, PAGE-purified by the manufacturer) (Supplemental Table S3). Oligonucleotide concentrations were quantified using A260 measurements on a NanoDrop spectrophotometer and extinction coefficients provided by the manufacturer. Ligation was performed using 2 nmol of each of the 5′ and 3′ RNA segments and splint in a combined volume of 60 µL to which 7.5 μL hybridization buffer (150 mM HEPES pH 7.5 and 500 mM KOAc) was added. This solution was heated for 2 min at 90°C and allowed to cool at room temperature for 30 sec before the 10× T4 RNA ligase 2 buffer (NEB) was added to a final concentration of 1×. The mixture was then cooled at room temperature for an additional 10 min, at which time additional ligase buffer, ATP, water, and T4 RNA ligase 2 (MCLabs) were added to achieve a reaction volume of 100 μL containing 0.4 mM ATP, 1× RNA ligase 2 buffer, and 100 units T4 RNA ligase 2. The ligation mixture was incubated at room temperature for 3 h. Following this, 16 units of DNase I (NEB) was added along with 10× DNase I buffer to bring the final concentration of that buffer to 1×. This mixture was incubated at 37°C for 15 min to digest the DNA splint.

A 2× FALB denaturing loading buffer consisting of 90% formamide (TCI) in 1× TBE was added to a final concentration of 1×, and the ligation mixture was heated for 2 min at 90°C to inactivate the enzyme and unfold the RNA. The resulting RNA mixture was run on a 12% polyacrylamide, 8 M urea denaturing gel and imaged using an Amersham Typhoon (GE). The gel was then stained with SYBR Gold in 1× TBE for 15 min, and the 76 nt band corresponding to the full-length tRNA was excised while visualizing the gel on a Dark Reader transilluminator (Clare Chemical Research). The RNA was extracted using an electro-elution system (Bio-Rad) run at 10 mA per fritted tube for 15 min in 0.5× TBE. After elution, the polarity was reversed for 1 min to release the RNA from the membrane, and the product was recovered from the apparatus. 1/10 volume of 3 M sodium acetate pH 5.5 and 2.5 volumes of −20°C 100% EtOH were added, and the solution was mixed and stored at −20°C overnight. Tubes were centrifuged at 4°C, 10,000 rcf for 40 min. The supernatant was removed, 500 μL of −20°C 70% EtOH was added, the tube was vortexed for 30 sec and then centrifuged at 4°C, for 10 min at the maximum RCF rating of the tube. The supernatant was again removed, and pellets were dried in a vacuum centrifuge at room temperature for 15 min until dry. The pellets were dissolved in 50 μL DEPC-treated H_2_O and quantified using a NanoDrop.

### Protein expression and purification

A pGEX-5X-3-ScPus4 backbone plasmid encoding GST-Pus4 with a Factor Xa cleavage site between the GST tag and Pus4 protein sequence (PDG322) was used (Supplemental Table S1). The GST-tagged Pus4 vector was transformed into *E. coli* BL21(DE3) competent cells (Thermo Fisher Scientific). A single transformant was inoculated to make a 50 mL overnight starter culture, which was then diluted into a 4 L culture grown until reaching OD_600_ = 0.80. Cultures were grown at 37°C in TB medium containing 100 µg/mL ampicillin and shaken at 225 rpm. Upon reaching the desired OD_600_, the 4 L culture was shifted to 16°C for 30 min. Protein expression was induced by adding IPTG (GoldBio) to a final concentration of 1 mM, and cells were grown overnight for ∼16 h at 16°C with continuous shaking at 225 rpm. Cells were harvested by centrifugation, and the cell pellets were resuspended in 30 mL of buffer A (50 mM Tris-HCl pH 7.5, 400 mM NaCl, 10 mM MgCl_2_, 10% glycerol, 0.5 mM EDTA, 5 mM BME, 1 tablet of Pierce EDTA-free Protease Inhibitor [Thermo Fisher Scientific]), divided into two 50 mL conical tubes, and then flash-frozen in liquid nitrogen before storage at −80°C. Each tube contained enough material for a single protein purification. All subsequent procedures were performed at 4°C unless otherwise stated.

Conical tubes were thawed on ice. The following reagents were added to achieve their final concentrations, respectively: lysozyme (Sigma-Aldrich) to 1 mg/mL, DNase (GoldBio) to 10 µg/mL, MgCl_2_ (Fisher) to 0.5 mM, and RNase A (Fisher) to 30 µg/mL. This cell suspension was incubated for 30 min. Cells were lysed via sonication using a sonicator (Branson Sonifier 450) with 30 sec-on/30 sec-off cycles (50% duty cycle, power level of 4), repeated three times. Insoluble material was removed by centrifugation for 30 min at 30,000*g* in a JA-20 rotor. After clarification, the remaining soluble material was incubated with 2 mL of a 50% slurry of Glutathione-Sepharose beads (Cytiva) that had been pre-equilibrated with buffer A for 1 h with end-over-end rotation at room temperature. After incubation, the protein-bound beads were isolated using centrifugation (5 min at 500*g*) and then washed with 20 mL of buffer A. This wash step was repeated for a total of five times. Protein was then eluted from the beads with 1 mL of freshly prepared elution buffer (50 mM Tris-HCl pH 7.6, 10 mM reduced glutathione) after incubating for 5 min at room temperature with end-over-end rotation. This elution step was repeated five times, and the elutions were pooled together (∼5 mL).

The eluate was concentrated to ∼1 mL final volume using a 30 kDa Vivaspin concentrator (GE Healthcare). Using the same concentrator, the sample underwent exchange into buffer B (50 mM Tris-HCl pH 7.5, 100 mM KCl) by adding 10 mL of buffer B and performing centrifugation to concentrate to ∼1 mL final volume. This was repeated two more times. These protein samples were aliquoted into Eppendorf tubes and flash-frozen in liquid nitrogen before storage at −80°C. The yield was quantified using A_280_ measurements on a NanoDrop spectrophotometer. Protein purity was determined using SDS-PAGE with samples collected across steps of the protein purification process (Supplemental Fig. S1A).

### Testing purified protein for RNase contamination

Approximately 1 µg of tRNA isolated from yeast was incubated with 0.4 µM of Pus4 in a 10 µL total volume for 1 h at room temperature. Control samples of tRNA without protein and tRNA plus protein without incubation were also prepared. Samples were combined with 10 µL of 2× RNA loading dye (NEB), heated at 70°C for 8 min on a heat block, and then loaded onto a fresh pre-run PAGE gel, along with RNA ladder, using an Apogee V16 gel system. After running the gel at 60 mA for ∼1.5 h, the gel was stained with SYBR Gold for 10 min at room temperature and imaged using an Amersham Typhoon (GE) to assess tRNA degradation (Supplemental Fig. S1B).

### Yeast growth conditions

*pus4*Δ (YDG127) cells were streaked out onto YPD agar media plates from a frozen −80°C stock and grown at 30°C for 2 days (Supplemental Table S1). Colonies were inoculated into 5 mL YPD media in culture tubes and grown at 30°C in a rolling drum overnight. In the morning, 400 μL of each culture was transferred to culture tubes with 5.2 mL liquid YPD and grown at 30°C in a rolling drum to an OD_600_ of ∼0.8. Cultures were transferred to 15 mL conical tubes and centrifuged at 4°C, 3000*g* for 2 min. Media was decanted, and the cell pellets were resuspended in 1 mL cold 1× PBS and transferred to microcentrifuge tubes. Tubes were centrifuged at 4°C, 9500 rpm for 1 min to pellet. Supernatant was decanted, and cell pellets flash frozen by placing microcentrifuge tubes in liquid nitrogen. Cell pellets were stored at −80°C until used for total RNA isolation.

### tRNA isolation from yeast

*pus4*Δ (YDG127) yeast pellets were thawed on ice (Supplemental Table S1). For cell lysis and RNA preservation, pellets were resuspended in 400 μL TES buffer (10 mM Tris, 10 mM EDTA, 20% SDS). Four hundred µL of acidic phenol was added to each tube. Tubes were vortexed for 10 sec and incubated in a 65°C water bath for 1 h, with 10 sec of vortexing every 15 min. Tubes were put on ice for 5 min and then centrifuged at 4°C, 13,000 rpm for 10 min. The top aqueous layer of each sample was transferred to a new tube. Four hundred µL of chloroform was added to each of the new tubes. Tubes were vortexed for 10 sec and then centrifuged at 4°C, 13,000 rpm for 10 min. The top aqueous layer of each sample was transferred to a new tube, and the addition of chloroform, vortexing, and centrifuging were repeated. The top aqueous layer of each sample was transferred to a new tube. To precipitate RNA, 1/10 volume of 3 M sodium acetate pH 5.5 and 2.5 volumes of ice cold 100% EtOH were added to each sample and mixed. Tubes were stored at −80°C for 1 h. Tubes were centrifuged at 4°C, 13,000 rpm for 10 min. The supernatant was removed, and 500 μL cold 70% EtOH was added to each tube to wash pellets of salt. Tubes were centrifuged at 4°C, 13,000 rpm for 10 min. Ethanol was removed, and tubes were left open for 5–10 min to allow residual ethanol to evaporate. Dried pellets were resuspended in 25 μL water, and their concentrations were quantified using a NanoDrop.

An 8% urea acrylamide gel was made using the National Diagnostics UreaGel 19:1 Denaturing Gel System. The gel was pre-run in 1× TBE running buffer at 45 mA for 30 min. Total RNA samples were aliquoted to 10 μL volumes, and 10 μL 2× RNA loading dye (NEB) was added to each sample. Three µL of Low Range ssRNA Ladder (NEB) was mixed with 7 μL water and 10 μL 2× RNA loading dye. Samples were incubated at 70°C for 8 min. Samples were loaded on the gel, and empty wells were loaded with 10 μL water + 10 μL 2× RNA loading dye. The gel was run at 20 mA for 15 min and then at 60 mA for ∼1 h. The gel was stained with SYBR Gold (Invitrogen) in 1× TBE running buffer for 10 min. The gel was imaged with an Amersham Typhoon (GE). Using UV shadowing, tRNA bands were cut from the gel. Gel fragments were placed in tubes with 450 μL 0.3 M NaCl. Tubes were put on a tube inverter overnight at 4°C. Liquid was transferred to new tubes, and 1.05 mL 100% EtOH was added to each sample and mixed. Samples were stored at −80°C for 1 h. Samples were centrifuged at 4°C, 13,000 rpm for 35 min. The supernatant was removed, and tubes were left open for 5–10 min to allow residual ethanol to evaporate. Dried pellets were resuspended in 10–20 μL water and stored at −80°C.

### In vitro modification

To perform the in vitro modification, 1 µM Pus4 protein was mixed with 300 nM total tRNA harvested from *pus4*Δ cells and incubated at 30°C for 2 min in a 200 μL reaction volume in buffer B (50 mM Tris-HCl pH 7.5, 100 mM KCl). Buffer B supplemented with 4 mM MgCl_2_ was also tested to observe whether magnesium affected catalysis. Since it did not have a strong effect (Fig. 2), MgCl_2_ was excluded from the buffer used for all smFRET and BLI assays. These reactions were halted with the addition of 200 µL of saturated phenol (pH 6.6). Samples were mixed and then stored at −80°C.

To purify in vitro modified tRNA, samples were thawed on ice, then vortexed for 10 sec, and centrifuged at 4°C, 13,000 rpm for 10 min. tRNA was repurified as done in the tRNA isolation (described above), beginning with the addition of 400 μL chloroform. Samples were resuspended in 10 μL water and concentrations quantified using a NanoDrop. Samples were stored at −80°C.

### Direct sequencing of in vitro modified tRNA

To perform library preparation for sequencing using Oxford Nanopore Technology (ONT), samples were first thawed on ice. Samples were incubated at 95°C for 2 min, room temperature for 2 min, and then on ice for 2 min. In a new tube, 7.25 μL tRNA sample was mixed with 2 μL 10× RNA ligase 2 buffer (NEB), 0.8 μL each of 10 μM ACCA, 10 μM GCCA, 10 μM UCCA, 5 μM CCCA, and 5 μM CCA specific adapters (Shaw et al. 2024), 2 μL 50% PEG, 2 μL 250 mM MgCl_2_, 1.25 μL 100 mM DTT, 2μL 20 mM ATP, and 1 μL RNA ligase 2 (NEB), for a total volume of 20 μL. The reaction was incubated at room temperature for 45 min. Twenty µL of water was added to the reaction. tRNA was purified with 1.8× volume RNAClean XP Beads (Beckman Coulter). Samples were washed twice with 500 μL 80% EtOH and eluted with 23 μL water. Samples were mixed with 8 μL 5× quick ligase buffer (NEB), 6 μL RMX adapter (ONT), and 3 μL T4 DNA ligase (2,000,000 U/mL; NEB) for a total volume of 40 μL. The reaction was incubated at room temperature for 30 min. tRNA was purified with 1.5× volume RNAClean XP Beads. Samples were washed twice with 150 μL WSB (ONT) and then eluted with 15 μL ELB (ONT). Water was added to bring the total volume to 37.5 μL. Samples were sequenced with MinION devices following the ONT SQK-RNA002 protocol.

Pseudouridylation of U_55_ in tRNA leads to a base miscall, due to an alteration in the ionic current when a pseudouridine transitions through the nanopore, as compared to the different disruption in ionic current caused by the unmodified uridine base. This causes a “mismatch” between the sequenced base and the unmodified reference base. The “reference match probability” refers to the probability that a given base will match the reference base. Therefore, a low reference match probability at position 55 is indicative of a pseudouridylated base. For more details, see Shaw et al. (2024).

Sequencing results were base-called using Guppy v3.0.3, aligned with BWA-MEM (-W 13 –k 6 –xont2d), and quality controlled with SAMtools (Q1). marginAlign was used to create alignment and error models, and marginCaller was used to calculate mismatch probabilities. Python was used to calculate the reference match probability—equal to a value of 1.0 minus the mismatch probability value—and generate heat maps. DRS data files are listed in Supplemental Table S4.

### Preparation of sucrose gradients

A 6× buffer B solution was prepared and then mixed with a 60% sucrose solution dissolved in water at the appropriate ratios to achieve five sucrose solutions of 10%, 15%, 20%, 25%, and 30%—each achieving a final concentration of 1× buffer B. Once prepared, 980 µL of the 30% sucrose solution was pipetted into Ultra-Clear centrifuge tubes (½ in × 2 in, Beckman Coulter 344057) and then allowed to freeze at −80°C for 20 min. The 25% solution was layered on top, allowed to freeze, and this was repeated for the remaining solutions. Any samples to be compared were run with the same batch of sucrose gradients to control for batch variation. Sucrose gradients were prepared ahead of time and stored at −80°C.

### Sucrose gradient ultracentrifugation

Gradients were thawed overnight at 4°C prior to use the next day. A 100 µL solution with 10 µM purified GST-Pus4 with and without 10 µM tRNA derived from YDG1 (Supplemental Table S1) was allowed to sit at room temperature for 30 min prior to loading onto a gradient. Gradients were balanced and then spun in an ultracentrifuge (Beckman Coulter Optima XPN-100) in a SW 55 Ti Swinging Bucket Rotor (Beckman Coulter) for 4 h at 55,000 rpm. Following ultracentrifugation, gradients were fractionated by hand, serially removing 200 µL from the top of the gradient and aliquoting into 24 tubes. (Note that the final fraction had a volume in the range of 200–400 µL, due to subtle differences in the total gradient volume and aspiration efficiencies of the dense sucrose mixture after spinning). Fractions were flash-frozen in liquid nitrogen prior to storage at −80°C.

Five nanomoles of each gel filtration chromatography-purified standard (β-amylase and carbonic anhydrase, Millipore Sigma) was combined and run together in a separate gradient.

### SDS-PAGE of sucrose gradients

Sucrose gradient fractions were mixed 1:1 with 2× Laemmli buffer (Bio-Rad) containing 5% v/v 2-mercaptoethanol, vortexed for several seconds, boiled on a heat block at 95°C for 10 min, then vortexed again, and briefly spun down to condense liquid. Equal volumes from each fraction were loaded across three 4%–15% PAGE gels (Bio-Rad). After electrophoresis in 1× SDS running buffer, gels were stained with GelCode Blue Stain Reagent (Thermo Fisher Scientific) to visualize protein abundance across the fractions.

### Biolayer interferometry (BLI)

BLI was performed using the biotinylated and fluorophore-labeled RNA substrates used for smFRET as well as versions of tRNA eMet lacking fluorophores. After the ligation steps described above, the RNA was diluted in buffer B to a concentration of 60 nM. The solution was heated to 90°C on a heat block for 2 min and then allowed to cool for 10 min at room temperature before placing it on ice. BLI measurements were performed at 30°C on a Gator Plus Biolayer Interferometer (Gator Bio) utilizing proprietary GatorOne software for data collection. Gator Bio streptavidin-coated glass probes were presoaked in buffer B for at least 20 min before collecting baseline signal for 300 sec. The probes were immersed in 60 nM biotinylated tRNA until an adequate shift (∼1.2 nm) in signal was observed (∼60 sec). The probes were then submerged in 10 nM biocytin (Cayman Chemical) in buffer B to quench the remaining streptavidin sites. This was followed by a second quenching in buffer B supplemented with 0.2% Tween-20, which further aided in reducing nonspecific protein binding. For this reason, buffer B supplemented with 0.02% Tween-20 (“buffer C”) was used for all the following steps. Loaded probes were then incubated in buffer C until baseline was achieved (∼300 sec). To measure the association of the protein with the tRNA, the loaded probes were then incubated in 200 nM of GST-Pus4 in buffer C for ∼2500 sec or more. To measure dissociation, the probes were then submerged in buffer C for the same amount of time as the association step. Reference probes were included in some runs to assess nonspecific protein binding and RNA dissociation from the probe.

### Analysis of BLI data

To estimate the kinetics of Pus4 binding to tRNA, we attempted to isolate the initial rate of association before pseudouridylation (in the case of tRNA that was not premodified) or refolding occurred. In isolation, this early phase of binding represents a reversible second-order reaction (RNA + Pus4 ⇋ RNA·Pus4). We approximated its kinetics as pseudo-first order in Pus4, as the protein was present in bulk solution while the quantity of tRNA was limited by the number of binding sites on the probe. This is expected to yield an exponential shape to the association curve (Eq. 1):

(1) $${\left[\mathrm{RNA}\cdot \mathrm{Pus}4\right]}_{t}={\left[\mathrm{RNA}\right]}_{0}(1-e^{-k_{\mathrm{obs}}t})$$

where the observed rate of the reaction is:

$$k_{\mathrm{obs}}=k_{\mathrm{on}}[\mathrm{Pus}4]-k_{\mathrm{off}}$$

At each time point *t*, [RNA]_0_ = [RNA·Pus4]_t_ + [RNA]_t_, and at the final time point, [RNA]_final_ = [RNA]_0_−[RNA·Pus4]_final_. Equation 1 thus becomes:

(2) $$\frac{{\left[\mathrm{RNA}\cdot \mathrm{Pus}4\right]}_{\mathrm{final}}-{\left[\mathrm{RNA}\cdot \mathrm{Pus}4\right]}_{t}}{{\left[\mathrm{RNA}\cdot \mathrm{Pus}4\right]}_{\mathrm{final}}}=e^{-k_{\mathrm{obs}}t}$$

To estimate *k*_on_ and *k*_off_, the association phases of the BLI curves were converted to fractional saturation (FS) using Equation 3, where *s*(*t*) is the shift at time *t* and *s*_final_ is the shift at the final time point of the association phase:

(3) $$\mathrm{FS}(t)=1-\frac{s_{\mathrm{final}}-s(t)}{s_{\mathrm{final}}}$$

Two methods were then used to fit the fractional saturation curve: (1) The early segment of the association phase over which ∼75% of the overall shift occurred was fitted with a single exponential (Eq. 4) or (2) a longer period of the association phase was fitted with the sum of two exponentials (Eq. 5). The first 1000 sec were used in the biexponential fits for tRNA without fluorophores due to the prominent downturn in the curves at 800 nM, while the entire association phase was fitted in all other cases. In Equation 5, the faster of the two rates was taken to be *k*_obs_.

(4) $$\mathrm{FS}(t)=y-a\cdot e^{-k_{\mathrm{obs}}t}$$

(5) $$\mathrm{FS}(t)=y-a_{1}\cdot e^{-k_{1}t}-a_{2}\cdot e^{-k_{2}t}$$

These fits were performed for BLI curves obtained at 50, 200, and 800 nM Pus4. *k*_obs_ was then plotted against [Pus4], and the slope of a linear fit yielded an estimate for *k*_on_. In principle, the *y*-intercept of the fit yields *k*_off_. However, determining it accurately would require measurements at lower [Pus4], conditions that reduce both the accuracy of the pseudo-first order kinetics approximation and the separation of the timescales on which Pus4 accumulates on the probe and on which it modifies and/or remodels the tRNA it has already bound to. Because *k*_obs_ was <0.01 sec^−1^ at 50 nM Pus4 (the lowest concentration tested) for all RNAs and fitting approaches, we can reasonably assume that *k*_off_ is less than that value. Given the values obtained for *k*_on_ (Supplemental Fig. S5), this constrains the dissociation constant *K*_*D*_ = *k*_off_/*k*_on_ to a value less than ∼130 nM.

### Electrophoretic mobility shift assay

The RNA was annealed as described above for BLI at a concentration of 300 nM. The RNA was then further diluted to 30 nM in buffer B and incubated with varying concentrations (0, 15, 30, 60, 120, 200, 400, 600, 800 nM) of Pus4 in a total volume of 10 μL for 30 min at room temperature. Each 10 μL solution was mixed with 10 μL of loading buffer (2× TBE, 40% glycerol). The mixtures were run on an 8% polyacrylamide native/nondenaturing gel at 300 V for 2.5 h at 4°C. The gel was prepared from 40% 19:1 acrylamide/bisacrylamide (Bio-Rad), TEMED (Thermo Fisher Scientific), and ammonium persulfate (Thermo Fisher Scientific) and pre-run in 1× TBE running buffer at 300 V for 90 min. The gel was imaged using an Amersham Typhoon (GE).

### Single-molecule FRET (smFRET)

RNA was annealed as described above at a concentration of 10 nM and then diluted to 10 pM in buffer B supplemented with 5 mM D-glucose (Sigma-Aldrich), 165 U/mL glucose oxidase from *Aspergillus niger* (Sigma-Aldrich), 2150 U/mL catalase from *Corynebacterium glutamicum* (Sigma-Aldrich), and 5 mM Trolox (Sigma-Aldrich) as an oxygen scavenging system (OSS). This OSS was used to visualize the RNA during immobilization due to its fast-acting capability. This mixture was flowed onto a slide that had been passivated with DDS (Sigma-Aldrich) as previously described (Hua et al. 2014), incubated with 0.2 mg/mL BSA (Thermo Fisher Scientific) for 5 min, 0.2% v/v Tween-20 for 10 min, and 0.2 mg/mL streptavidin (Thermo Fisher Scientific) for 5 min. Imaging was performed with buffer C containing 5 mM 3,4-dihydroxybenzoic acid (Sigma-Aldrich), 5 mM Trolox (Sigma-Aldrich), and 50 nM protocatechuate 3,4-dioxygenase from *Pseudomonas* sp. (Sigma-Aldrich or MP Biomedicals) as an OSS and, for experiments with protein, 200 nM Pus4. This imaging buffer was incubated in a tube with minimal headspace for 10 min to allow the OSS to begin depleting oxygen before injection into the sample chamber. Data were collected at room temperature on a home-built prism-type TIRF microscope (Leica DMi8 Inverted Microscope, 63× oil immersion objective). Cy3 and Cy5 were excited by a 532 nm laser (Coherent OBIS 532 LS) at 140 mW and a 637 nm laser (Coherent OBIS 637 LX) at 60 mW, respectively. The slide was illuminated at 532 nm continuously and at 637 nm at the beginning and end of each movie to confirm that Cy5 was active. Two-minute-long movies were recorded on an Andor iXon 888 EMCCD with an exposure time of 100 msec. An EM gain setting of 300 was used for movies recorded in the presence of Pus4. tRNA-only movies used for intensity comparison (Supplemental Table S2) were recorded at the same EM gain, whereas tRNA-only movies used for FRET analysis (Fig. 5; Supplemental Fig. S12) were recorded at an EM gain of 450 due to lower fluorophore brightness in the absence of protein. Movies were collected back-to-back for the first 15 min following injection of imaging buffer, and additional movies were collected 30–34 min after injection. Unless otherwise noted, data reported for tRNA in the absence of Pus4 were recorded 10–15 min following injection of imaging buffer.

Coordinates for each molecule were extracted from raw video files using FIJI, and fluorophore intensity traces were extracted using custom MATLAB code. Traces that exhibited single-step photobleaching of Cy3 and Cy5 and anticorrelation between donor and acceptor signals when transitions occurred were selected manually and analyzed using custom MATLAB codes. FRET efficiency was calculated from the measured donor and acceptor emission intensities (*I*_*D*_ and *I*_*A*_) using Equation 6:

(6) $$E_{\mathrm{FRET}}=\frac{I_{A}-x\cdot I_{D}}{\gamma I_{D}+(I_{A}-x\cdot I_{D})}$$

Variable *x* corrects the acceptor signal for bleedthrough of donor emission into the acceptor channel and was determined to be 0.129 through analysis of tRNA molecules that lacked an active Cy5. γ was determined through analysis of traces in which Cy5 photobleached before Cy3, as previously described (McCann et al. 2010), and was found to be 2.42 in the absence of protein and 3.01 in the presence of protein (Supplemental Fig. S8C,D). Noise in the corrected donor and acceptor traces was suppressed using a bilateral filter with a spatial spread of two frames and a signal value spread of ∼1/4 of the mean signal intensity across all traces in a data set for Cy3 and ∼1/5 of the mean signal intensity for Cy5. This resulted in signal value spreads of 900–1000 for Cy3 and 800–1100 for Cy5 in the absence of protein and spreads of 2200–3100 for Cy3 and 1900–2500 for Cy5 in the presence of protein, which preserved rapid excursions in the resulting *E*_FRET_ traces.

FRET histograms were compiled from the first 50 frames of ∼200 traces and fitted with a sum of three or four Gaussian functions in Mathematica (Wolfram Alpha). Hidden Markov modeling was performed in Quantifying unknown Biophysics (QuB) Online (Milescu et al. 2000; Qin 2004; Nicolai and Sachs 2013), and TODPs were generated from the resulting idealized traces using custom MATLAB codes. Correlation analysis was performed in Mathematica by computing the cross-correlation *C*(τ) between Cy3 and Cy5 intensities using Equation 7 (Kuzmenkina et al. 2005):

(7) $$C(\tau )=\frac{<\mathrm{\Delta Cy}3(t)\mathrm{\Delta Cy}5(t+\tau )>}{<\mathrm{Cy}3(t)><\mathrm{Cy}5(t+\tau )>},$$

where Δ*X*(*t*) = *X*(*t*) − <*X* > and <*X* > is the average value of *X* across all frames *t* within the trace. For autocorrelation analysis, Cy3 and Cy5 intensities in the above equation were both replaced by the *E*_FRET_ value. Traces used for correlation analysis were corrected for bleedthrough and γ factor but not subjected to the bilateral filter. Autocorrelation and cross-correlation curves were fitted simultaneously with a two-exponential model (Eqs. 8 and 9, respectively) with the time constants *t*_1_ and *t*_2_ fixed across the two curves.

(8) $$\mathrm{Fit}(\tau )=a+b_{1}\cdot e^{-\tau /t_{1}}+b_{2}\cdot e^{-\tau /t_{2}}$$

(9) $$\mathrm{Fit}(\tau )=a_{2}+b_{3}\cdot (1-e^{-\tau /t_{1}})+b_{4}\cdot (1-e^{-\tau /t_{2}})$$

HMM-derived rate constants for transitions between FRET states were determined by extracting the durations of dwells in each state and fitting their cumulative distribution functions with Equation 9, where *k*_*i*_ = 1/*t*_*i*_.

### Bulk emission spectroscopy

Emission spectra were recorded on an Edinburgh Instruments FS5 Spectrofluorometer using a 4 mm square cuvette. RNA was annealed as described above at a concentration of 150 nM in buffer B and used without further dilution. Cy3 was excited at 532 nm with a bandwidth of 2 nm, and emission was scanned from 500 to 800 nm in 1 nm steps with a bandwidth of 2 nm and a 1 sec dwell time.

### Free energy calculations

The free energies of different secondary structures were predicted using the RNAfold web server (Gruber et al. 2008). The sequence used for mt-tRNA^Lys^ was the one utilized in Voigts-Hoffmann et al. (2007), with U instead of dT at its fluorophore-labeling sites, and the sequence used for tRNA eMet was the one utilized in this work. Mild base-pairing constraints were applied as needed to achieve the C and E secondary structures of mt-tRNA^Lys^ as depicted in Fig. 1 of Voigts-Hoffmann et al. (2007), the canonical tRNA eMet and tRNA Thr structures as depicted in Figure 1A of this work, and plausible E-like structures of tRNA eMet and tRNA Thr as depicted in Supplemental Figure S13B and D. The secondary structures were optimized under these constraints with energy parameters rescaled to 25°C and 0.1 M salt and isolated base pairs allowed. The resulting minimum free energy structures were re-rendered using R2DT (McCann et al. 2025) to generate the visualizations shown in Supplemental Figure S13.

## DATA DEPOSITION

The direct RNA sequencing data are publicly accessible at the European Nucleotide Archive https://www.ebi.ac.uk/ena (accession number PRJEB85651).

## SUPPLEMENTAL MATERIAL

Supplemental material is available for this article.
